# Book Reviews

**Published:** 1895-04

**Authors:** 


### *BOOK REVIEWS.*

A Manual of Diseases of the Ear. For Students and Practitioners of Medicine. By Albert H. Buck, M.D., Clinical Professor of Diseases of the Ear, College of Physicians and Surgeons, New York. Wm. Wood & Co., Publishers.

This volume is a second revised edition of Dr. Buck's most excellent work on the ear, which was first published in 1880 and again revised in 1889. It is unnecessary to call the reader's attention to the merits of this book if it has once been his pleasure and profit to peruse its pages. We have no hesitancy in saying that for practitioners of medicine, young aurists, and students it is the best work upon the ear published in the English language. This statement is made by no means superficially, since it has been our pleasure to examine into at least the most noteworthy works upon this subject. Dr. Buck is what you might term a "conservative aural surgeon," and therefore his views as set forth in this work are sure guides to follow. He is a surgeon of large clinical experience, and it is this clinical and practical manner in which his book is written which gives it the chief charm and utility. In this edition he has added one new chapter entitled Analysis of Symptoms, which is exceedingly clear and practical. The chapter of Diseases of the Mastoid has been somewhat enlarged and rewritten, setting forth clearly the author's views upon this important subject. Dr. Buck is not an advocate of excision of the drum and ossicles in diseases of the middle ear, and the sentence below stands in quite contradistinction to some found in Dr. Dench's late work on the ear, which was reviewed in the last issue of this Journal: "The question of treatment of these deformities of the middle ear may be dismissed in a very few words. Excision of the hammer and attempts to render it mobile by the employment of a certain degree of force in cases where the ossicle has acquired a rigid, immovable position do not commend themselves to my judgment. Operative interference in cases of adhesions between the membrana tympani and the mucous membrane of the promontory, or between the ossicles and some portions of the tympanum, may possibly, in certain cases, accomplish some good, but, as already stated, I have only encountered one case in which I felt the slightest degree encouraged to undertake such an operative interference." This work of Dr. Buck is unusually clear and explicit, and its teachings can be followed with safety throughout. R.

---

MEMORANDA AND TABLES OF HUMAN ANATOMY. Vol. I. Being a complete description of the Muscles, Ligaments, etc. For the Use of Practitioners and Students of Medicine. By Justin Herold, A.M., M.D. Formerly House Physician and Surgeon to St. Vincent's Hospital, etc., etc., etc., and Sebastian J. Wimmer, M.A., M.D., author of Tables and Notes on Human Osteology; The Physician's Vade Mecum, etc., etc., etc. With a Preface by Professor James A. Garretson, A.M., M.D. Philadelphia: The Medical Publishing Co., 718 Betz Building. Price, $1.50.

This volume treats of the Muscles, Ligaments, Synovial Membranes, and Articulations. The book is small, neat in appearance and compact. The arrangement is the best we have ever seen. The muscles are described by regions, the name of each region being bracketed opposite the names of the muscles belonging there. The list having been completed, the description of each muscle (by regions) is taken up. To give an idea of the arrangement, it suffices to copy from one brief description, as follows:

13. PYRAMIDALIS NASI.

*Description.* { Small and pyramidal, with its summit upward.

*Origin.* { Is continuous with the occipito-frontalis above.

*Insertion.* { Descending vertically, it terminates at the root of the nose, blending with the compressor nasi.

*Actions.* { The skin of the root of the nose is wrinkled, etc.

*Relations.* { (a) *Upper surface* with the skin.

(b) *Under surface* with frontal and nasal bones.

*Nerve.* { Facial.

The other subjects are arranged in as convenient manner.

M. B. H.

TEXT-BOOK OF NORMAL HISTOLOGY, Including an Account of the Development of the Tissues and Organs. By George A. Piersol, M.D., Professor of Anatomy in the University of Pennsylvania. With four hundred and nine Illustrations, of which three hundred and fifty-eight are from original drawings, by the author. Third edition. Philadelphia. J. B. Lippincott & Co. 1895.

This is a book of some four hundred pages. It is the work of an American author which, other things being equal, should be in its favor. The illustrations are good. A few of these appear obscure at first sight, but this is only an evidence of their accuracy, because we often find that the best microscopic sections are very much less clear than pictures representing them. Often the illustrations show a single field typically, then a composite of many fields, to give the whole structure. The text is rather full for one who would begin the study of histology, but one who begins the study of anatomy with Gray will be able to endure that.

The descriptions of the tissues and organs are given with great thoroughness. Their development is also described. Unfortunately for those who do not understand it, only the metric system is used. The English should have been used in parenthesis to guide the numerous students who have no knowledge of the metric standards.

At the end is an appendix, in which the best histological methods are given.

The work deserves to take a leading place as a text-book of histology. M. B. H.

---

A MANUAL OF BANDAGING, Adapted for Self-instruction. By C. Henri Leonard, A.M., M.D., Professor of the Medical and Surgical Diseases of Women, and Clinical Gynecology in the Detroit College of Medicine. Sixth edition, with 139 engravings. Cloth, octavo, 189 pages. Price, $1.50. The *Illustrated Medical Journal* Co., Publishers, Detroit, Mich.

The main feature for commendation of this book over other similar works is that each illustration shows the direction of the various turns of the bandage with arrow-heads, and each turn is properly numbered; this renders the book a self-instructor to the reader of it, who has but to put the various bandages about the limbs of an office companion a few times, when the "trick" of its application upon a patient has been learned. It takes the place, in this way, of hospital drill. Besides the "Roller Bandages," the various

"Slings," "Tailed," "Adhesive" and "Plaster" bandages, and "Immovable Dressings" are given. The book is divided into sections, treating of "The Bandages of the Head," of "The Body," of "The Upper Extremity," of "The Lower Extremity," "Knots," "Strappings," "Compresses," and "Poultices," with full description of making and applying the same. There is an illustration for nearly every bandage described. A medical student could profitably spend his vacation evenings in mastering the application of bandages by using this book as a guide; and to a practitioner it would not come amiss.

---

SURGICAL PATHOLOGY AND THERAPEUTICS. By John Collins Warren, M.D., Professor of Surgery in Harvard University; Surgeon to the Massachusetts General Hospital. Illustrated. Price, $6.00. Published by W. B. Saunders, 925 Walnut St., Philadelphia.

The mission of this work is to supply the student and surgeon with the scientific portion of a surgical education. No one will gainsay the importance of such knowledge. The best surgeon must understand the principles as well as the actual practice of surgery. The present volume is a splendid success in this direction. It very properly begins with a thorough and complete discussion of bacteriology and surgical bacteria (78 pages). The next hundred pages describe the phenomena of simple and infective inflammations. The next chapters deal with gangrene, shock, the surgical fevers, septicemia, pyemia, erysipelas, tetanus, tuberculosis of the bones, joints, and soft parts, etc. One hundred and fifty pages are devoted to the tumors. The last chapter, twenty-five pages, describes the principles of aseptic and antiseptic surgery. So far as we know, this is the only American work of this class which can lay any claim to completeness. It will be better adapted for us, therefore, than the German volumes which we have heretofore depended upon. The book is plentifully illustrated and excellently printed. We heartily recommend it. It should reach a wide circulation among American readers.

---

A PRACTICAL THEORY AND TREATMENT OF PULMONARY TUBERCULOSIS, by Frank S. Parsons, M.D., editor of the Philadelphia *Medical Times and Register*. Published by the Medical Publishing Company, 718 Betz Building, Philadelphia, Pa. Price, 25 cents. Paper cover.

This monograph covers seventy-seven pages of a neat little volume. It treats of a subject of universal interest to all scientifically inclined persons.

The first pages are devoted to an interesting introductory, illustrative of the present condition of medical thought upon the subject. The causation of tuberculosis is then taken up, and the author endeavors to show that the dominant theory regarding the tubercle bacillus as a causative agent is not based on the true pathological condition in the early stage of phthisis. Bacilli are to be regarded only as developments, existing because a favorable medium presents. This medium exists before the bacillus is demonstrable, and consists of the waste elements of the blood congregating in a locality through lymphatic obstructions or stasis.

The author thinks that a diseased pancreas probably plays a more important part in the etiology and symptomatology of phthisis than is generally supposed. The book will be found very readable.

MANUAL OF CHEMISTRY. A Guide to Lectures and Laboratory work for Beginners in Chemistry. A Text-book specially adapted for Students of Pharmacy and Medicine. By W. SIMON, Ph.D., M.D., Professor of Chemistry and Toxicology, College of Physicians and Surgeons, Baltimore; Professor of Chemistry in the Maryland College of Pharmacy. New (fifth) edition. Cloth, $3.25. Philadelphia: Lea Brothers & Co.

The exhaustion of the large fourth edition of Professor Simon's Chemistry in less than two years indicates something of the position and popularity which it has achieved as a text-book in medical and pharmaceutical colleges. It is therefore too well known to require extended notice here. For this edition proper revisions and additions have been made in accordance with late advances in chemistry and pharmacy, and in order to bring it in harmony with the new Pharmacopeia. An important feature is the careful description of modern methods for chemical examination in clinical diagnoses. There are forty-four engravings and eight handsome colored plates, illustrating sixty-four important chemical reactions and tests. This fifth edition of this good work will be even more useful than its predecessors.

WE have received from the J. B. Lippincott Company, Philadelphia, an excellent little volume entitled "Suggestions to Hospital and Asylum Visitors," by Dr. John S. Billings and Dr. H. M. Hurd, with an Introduction by Dr. S. Weir Mitchell. It has been prepared as a guide for those whose duties (non-professional) call them to visit or inspect the various departments of hospitals and asylums. To such it will be very useful, teaching them how to understand and appreciate the details of hospital management and technique.

